# Efficacy of Incremental Next-Generation ALK Inhibitor Treatment in Oncogene-Addicted, *ALK*-Positive, *TP53*-Mutant NSCLC

**DOI:** 10.3390/jpm10030107

**Published:** 2020-08-28

**Authors:** László Urbán, Róbert Dóczi, Barbara Vodicska, Dóra Kormos, László Tóth, István Takács, Edit Várkondi, Dóra Tihanyi, Dóra Lakatos, Anna Dirner, István Vályi-Nagy, István Peták

**Affiliations:** 1 Department of Pulmonology, Mátraháza University and Teaching Hospital, 3233 Mátraháza, Hungary; tothrad@gmail.com; 2 Oncompass Medicine Hungary Ltd., 1024 Budapest, Hungary; robert.doczi@oncompassmedicine.com (R.D.); barbara.vodicska@oncompassmedicine.com (B.V.); edit.varkondi@oncompassmedicine.com (E.V.); dora.tihanyi@oncompassmedicine.com (D.T.); dora.lakatos@oncompassmedicine.com (D.L.); anna.dirner@oncompassmedicine.com (A.D.); 3 Department of Internal Medicine and Lymphomatherapy V., BAZ County Central and Teaching Hospital, 3526 Miskolc, Hungary; kormosdora92129@gmail.com (D.K.); stevenweavermd@gmail.com (I.T.); 4 Faculty of Healthcare, University of Miskolc, 3515 Miskolc, Hungary; 5 National Hematology and Infectology Institute, Centrum Hospital of Southern Pest, 1097 Budapest, Hungary; drvnistvan@gmail.com; 6 Department of Pharmacology and Pharmacotherapy, Semmelweis University, 1089 Budapest, Hungary; 7 Department of Pharmaceutical Sciences, University of Illinois at Chicago, Chicago, IL 60612, USA

**Keywords:** NSCLC, *ALK* rearrangement, *TP53* mutation, TKI, personalized treatment

## Abstract

Background: The anaplastic lymphoma kinase (*ALK*) gene fusion rearrangement is a potent oncogene, accounting for 2–7% of lung adenocarcinomas, with higher incidence (17–20%) in non-smokers. *ALK*-positive tumors are sensitive to ALK tyrosine kinase inhibitors (TKIs), thus *ALK*-positive non-small-cell lung cancer (NSCLC) is currently spearheading precision medicine in thoracic oncology, with three generations of approved ALK inhibitors in clinical practice. However, these treatments are eventually met with resistance. At the molecular level, *ALK*-positive NSCLC is of the lowest tumor mutational burden, which possibly accounts for the high initial response to TKIs. Nevertheless, *TP53* co-mutations are relatively frequent and are associated with adverse outcome of crizotinib treatment, whereas utility of next-generation ALK inhibitors in *TP53*-mutant tumors is still unknown. Methods: We report the case of an *ALK*-positive, *TP53*-mutant NSCLC patient with about five years survival on ALK TKIs with continued next-generation regimens upon progression. Results: The tumor showed progression on crizotinib, but long tumor control was achieved following the incremental administration of next-generation ALK inhibitors, despite lack of evident resistance mechanisms. Conclusion: *TP53* status should be taken into consideration when selecting ALK-inhibitor treatment for personalized therapies. In *TP53*-mutant tumors, switching TKI generations may overcome treatment exhaustion even in the absence of ALK-dependent resistance mechanisms.

## 1. Introduction

Fusion rearrangements of the anaplastic lymphoma kinase (*ALK*) gene lead to dimerization and constitutive activation of the encoded tyrosine kinase and thus the downstream transforming signaling pathways [1]. *ALK* fusions account for approximately 2–7% of patients with lung adenocarcinoma, with this frequency being higher, 17–20% within non-smoker patients [2]. *ALK*-positive non-small-cell lung cancer (NSCLC) is currently spearheading the advent of precision medicine in thoracic oncology. At the molecular level, this is attributable to the lowest tumor mutational burden, with few co-occuring mutations, and to the lowest frequency of *TP53* (encoding cellular tumor antigen p53) mutations (20–25%) among NSCLCs [3].

*ALK*-positive tumors are sensitive to small-molecule ALK tyrosine kinase inhibitors (TKIs). The FDA approved the first-generation inhibitor crizotinib as a monotherapy for the treatment of *ALK*-positive metastatic NSCLC patients based on the results of two clinical trials. As first-line therapy, crizotinib was shown to be superior to standard chemotherapy (pemetrexed and platinum) in 343 advanced *ALK*-positive NSCLC patients, in an open-label, phase 3 trial (NCT01154140). The objective response rate (ORR) was 74% versus 45% in the crizotinib and the chemotherapy-receiving groups, respectively. Statistically significant improvement was obtained in the median progression-free survival (PFS) in response to crizotinib compared with chemotherapy (10.9 versus 7.0 months; hazard ratio (HR): 0.45; 95% confidence interval (CI): 0.35–0.60; *p* < 0.001) [4]. The median overall survival (OS) was not reached (NR) in the crizotinib arm (95% CI: 45.8 months to NR) and was 47.5 months in the chemotherapy arm (95% CI: 32.2 months to NR) [5].

Second-generation ALK inhibitors, ceritinib, alectinib, and brigatinib, proved to be active in patients with tumor progression on crizotinib [6]. Ceritinib was also approved by the FDA for the treatment of *ALK*-positive metastatic NSCLC patients based on the outcomes of two clinical trials. In the ASCEND-4 randomized, open-label, phase 3 study (NCT01828099), the efficacy of ceritinib versus platinum-based chemotherapy (cisplatin or carboplatin and pemetrexed) was evaluated in advanced *ALK*-positive NSCLC patients (n = 376). The overall response rate was 73% (95% CI: 66–79%) versus 27% (95% CI: 21-34%), the median PFS was 16.6 months (95% CI: 12.6–27.2) versus 8.1 months (95% CI: 5.8-11.1) (HR: 0.55; 95% CI: 0.42–0.73; *p* < 0.00001) in the ceritinib group compared with the chemotherapy-receiving group, respectively. The median OS was not reached in the ceritinib group (95% CI: 29.3 months to NR) and was 26.2 months (95% CI: 22.8 months to NR) in the chemotherapy group (HR: 0.73; 95% CI: 0.50–1.08; *p* = 0.056) [7].

In the ASCEND-1 open-label, phase 1 trial (NCT01283516), the efficacy of ceritinib was tested among *ALK*-positive NSCLC patients, both in the ALK-inhibitor naive (n = 83) and crizotinib-pretreated (n = 163) setting. The overall response rate was obtained as 72.3% (95% CI: 61.4–81.6%) and 56.4% (95% CI: 48.5–64.2%), median PFS was 18.4 months (95% CI: 11.1 months to NR) and 6.9 months (95% CI: 5.6–8.7 months), and median OS was not reached (95% CI: 19.6 months to NR) and 16.7 months (95% CI: 14.8 months to NR) in the ALK-inhibitor naive and pretreated groups, respectively [8].

Lorlatinib is a third-generation, reversible, ATP-competitive inhibitor of ALK and ROS1 (Proto-oncogene tyrosine-protein kinase ROS), which can also penetrate the blood–brain barrier and overcome known *ALK* resistance mutations [9]. The FDA approved lorlatinib for *ALK*-positive metastatic NSCLC patients whose disease progressed on at least one previous ALK-inhibitor therapy, based on a non-randomized, dose-ranging phase 2 study (NCT01970865). Of the 276 enrolled patients, 229 were *ALK*-positive, among them 30 were treatment-naive (EXP1), 59 patients received crizotinib without (n = 27; EXP2) or with chemotherapy (n = 32; EXP3A), 28 received one previous non-crizotinib ALK inhibitor, with or without chemotherapy (EXP3B), 66 received two (EXP4) and 46 received three (EXP5) previous ALK inhibitors with or without chemotherapy. In treatment-naive patients (EXP1) the ORR was 90% (95% CI: 73.5–97.9%), in patients with at least one previous ALK-inhibitor therapy (EXP2-5) the ORR was obtained as 47.0% (95% CI: 39.9–54.2%). In the EXP2-3A, EXP3B, and EXP4-5 subgroups, ORR values were 69.5% (95% CI: 56.1–80.8%), 32.1% (95% CI: 15.9–52.4%) and 38.7% (95% CI: 29.6–48.5%), respectively. In treatment-naive patients (EXP1) and ALK-inhibitor pretreated patients (EXP2-5) the median PFS values were obtained as NR (95% CI: 11.4 months to NR) and 7.3 months (95% CI: 5.6–11.0 months), respectively. In the EXP2-3A, EXP3B, and EXP4-5 subgroups, median PFS was NR (95% CI: 12.5 months-NR), 5.5 months (95% CI: 2.7–9.0 months), and 6.9 months (95% CI: 5.4–9.5 months), respectively. OS results were not published [10].

Despite sensitivity to ALK TKIs, relapse or tumor progression is systematically noted, as tumor evolution invariably leads to acquired resistance to ALK TKIs [1,6,11]. Resistance mechanisms are driven by two distinct underlying processes—ALK-dependent mechanisms, such as secondary resistance mutations or amplification of *ALK*; and ALK-independent mechanisms, leading to activation of signaling pathways so tumor cells are enabled to escape ALK dependency [1]. It is generally viewed that secondary *ALK* mutations indicate continued ALK dependency and sensitivity to ALK TKIs with activity against the resistance mutation, whereas in the absence of resistance mutations, ALK-independent mechanisms give rise to resistance and thus combinatorial treatments or standard therapy approaches should be considered.

Despite the relatively low mutational burden of *ALK*-positive tumors, *TP53* co-mutations occur relatively frequently and they have been recently identified as main molecular determinants of adverse outcome, representing a negative prognostic factor for PFS and OS [12,13]. These studies focused on crizotinib and thus the utility of next-generation ALK inhibitors in *TP53*-mutant *ALK*-positive tumors is still to be addressed. Moreover, it is long established that *TP53* mutations can affect chemotherapy treatments [14,15] and they are negative prognostic factors for chemotherapy in *ALK*-rearranged NSCLC [16].

**Figure 1:**
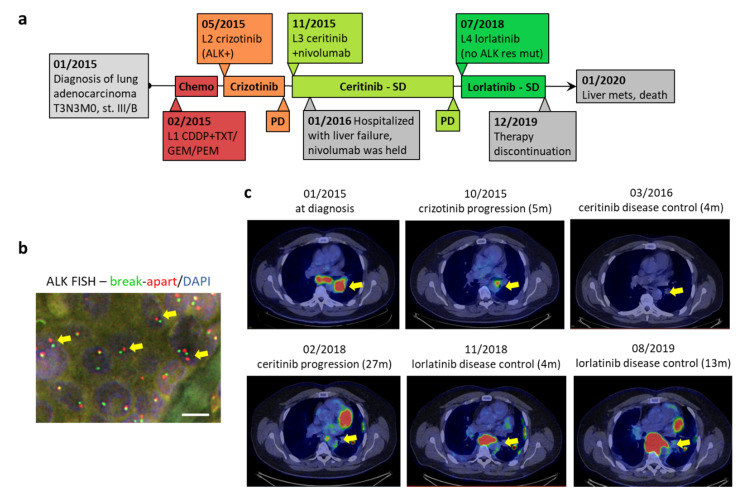
Case representation. (**a**) Time course of the case presented. First line chemotherapy of cisplatin+docetaxel (CDDP + TXT), then cisplatin + gemcitabine/pemetrexed (CDDP + GEM/PEM) was changed to 1st generation anaplastic lymphoma kinase (ALK) inhibitor after molecular diagnostic results. Following fast progression on crizotinib, a 2nd generation ALK inhibitor, ceritinib was started in combination with nivolumab. Due to liver damage, nivolumab was stopped two months later. Ceritinib resulted in disease control for 2.5 years. Upon progression, with no ALK resistance mutations detected, a 3rd generation ALK inhibitor, lorlatinib, controlled disease progression for 1.5 years until the patient stopped taking medication, that ultimately led to a fast development of fatal liver metastases. L1-4—treatment lines. PD—progressive disease. SD—stable disease. Mets—metastases. (**b**) Representative image of *ALK* FISH analysis. Yellow arrows at split signals indicate *ALK* rearrangement. Nuclei are stained with DAPI (blue). Scale bar: 5 µm. (**c**) Representative PET-CT slices showing pulmonary tumor mass in different time points. Numbers in brackets indicate months on treatment. Yellow arrow indicates tumor mass.

Here we present an interesting case of a *TP53*-mutant, *ALK*-positive NSCLC patient, who was resistant to crizotinib, but responded to next-generation ALK inhibitors in the absence of ALK-dependent resistance mechanisms (Figure 1a).

## 2. Case Report

In December 2014, a 50-year-old male never-smoker showed up at a medical examination with increasing cough over the last five months. The cough had recently become productive, and the sputum was occasionally red. Chest CT scan, bronchoscopy and positron emission tomography–computed tomography (PET-CT) confirmed stage III lung adenocarcinoma in the left lower lobe with mediastinal and hilar lymph node involvement on both sides (T3N3M0, stage III/B) (Figure 1c). Below the affected region he developed atelectasis. In January 2015, the patient underwent mediastinoscopy. Pathology test results showed that the lymph node metastasis was negative for *EGFR* (Epidermal growth factor receptor) and *KRAS* (GTPase KRas (Kirsten rat sarcoma)) mutations and *ALK* rearrangement.

In February, treatment with cisplatin and docetaxel (75 mg/m^2^ each) was commenced, but due to an allergic reaction to taxol, they were switched to cisplatin (75 mg/m^2^) + gemcitabine (1200 mg/m^2^)/pemetrexed (500 mg/m^2^). In March 2015, sampling of the primary tumor was successful with explorative thoracotomy and the tumor turned out to be inoperable. Histopathologic examination of formalin-fixed paraffin-embedded (FFPE) block section of the tissue revealed positivity for *ALK* in 56% of cells (63/113) using two channels of the ZytoLight SPEC ALK/EML4 TriCheck Probe specific for *ALK* (Figure 1b). The fusion partner has not been determined. Fluorescence in situ hybridization (FISH) was negative for *HER2* (Receptor tyrosine-protein kinase erbB-2 (human epidermal growth factor receptor 2)), *MET* (Hepatocyte growth factor receptor), *FGFR1* (Fibroblast growth factor receptor 1), and *PIK3CA* (Phosphatidylinositol 4,5-bisphosphate 3-kinase catalytic subunit alpha isoform) amplification and *ROS1* rearrangement. Next-generation sequencing of 50 genes (50-gene Cancer HotSpot Ampliseq panel, Thermo Fisher) detected two missense mutations: R273H (c.818G > A) in exon #7/10 of *TP53*, which hits the DNA-binding domain of p53, and Q472H (c.1416A > T) in exon #11/30 of *KDR* (encoding vascular endothelial growth factor receptor 2 (VEGFR-2)), which hits the immunoglobulin-like C2-type 5 domain within the extracellular region of VEGFR-2.

In the light of the findings, from May 2015, crizotinib (first generation ALK inhibitor) therapy was started (2 × 250 mg (MD 250 mg)). After five months on crizotinib, PET-CT demonstrated progression on the primary left lower lobe tumor and on the mediastino-hilar lymph nodes (Figure 1c). Novel metastatic mass was not detected. Due to progression, in November 2015, his treatment was changed to the second-generation ALK inhibitor ceritinib (450 mg/day) plus nivolumab (240 mg biweekly). Two months after the second administration of nivolumab, the patient was hospitalized with severe liver failure demonstrated by subicterus and elevated liver enzymes (alkaline phosphatase (AP) values over 3000 U/L) and a C-reactive protein (CRP) value of 175 mg/L. Endoscopic retrograde cholangiopancreatography (ERCP) revealed cholelithiasis and immune-related hepatitis. Nivolumab was held. Ceritinib therapy (450 mg/day) was continued and resulted in stable disease for over 2.5 years without metastatic lesions. In June 2018, PET-CT revealed morphometabolic progression of the mediastino-hilar lymph nodes (Figure 1c).

Therefore, in July 2018, therapy was switched to the third-generation ALK inhibitor, lorlatinib (100 mg/day), which stabilized the disease (Figure 1c). ALK inhibitor resistance mutations (C1156Y, I1171N, L1196M, G1202R, or G1269A) were not detected by droplet digital PCR (ddPCR) on liquid biopsy. After showing no signs of progression or metastatic lesions for 17 months on lorlatinib, the patient arbitrarily discontinued medication without informing his physician. One month later, in January 2020, he was hospitalized with severe liver failure, which turned out to be the consequence of multiple liver metastases. The patient’s health condition and organ function could not be improved due to the advanced tumor stage and he succumbed to the disease one week after admission to the hospital.

## 3. Discussion

Here we report the case of an *ALK*-positive, *TP53*-mutant NSCLC patient with about five years survival on ALK-inhibitor TKIs with continued next-generation regimens upon progression.

Non-small-cell lung cancer encompasses a wide spectrum of molecular subtypes by driver mutations. The *EML4-ALK* gene fusion is the consequence of a paracentric inversion of chromosome two, which was first reported in NSCLC in 2007 [17]. The never-smoker history of the patient is in line with *ALK* fusions being more prevalent in the non-smoker NSCLC subpopulation. Moreover, *ALK* positivity is a biomarker of poor prognosis in a population of non-smoker patients [18], further highlighting the importance of personalized treatment.

The development of three generations of ALK TKIs has substantially changed the landscape for the treatment of *ALK*-positive tumors [1,19], as the OS of patients with *ALK*-rearranged NSCLC is five years [20]. Yet, despite frequent long-lasting responses to ALK TKIs, resistance to these drugs almost inevitably occurs [1,20]. ALK TKI resistance may emerge either in an ALK-dependent fashion (i.e., secondary resistance mutations or amplification of *ALK*) or ALK independently (i.e., activating downstream mutations, such as *KRAS* or *BRAF*) whereby tumor cells escape ALK dependency [1]. During the course of the disease, progression developed at each line of systemic therapy, yet successive next-generation ALK TKIs demonstrated disease control at each consecutive line.

The rapid and dramatic progression of the disease immediately after the patient quit TKI treatment clearly indicates oncogene addiction, implying the absence of ALK-independent resistance mechanisms. The notion of oncogene addiction is further underscored by the failure of nivolumab combination therapy. Nevertheless, liquid biopsy tests were negative for the common secondary *ALK* mutations, suggesting that disease progression was probably not caused by resistance mutations either. Therefore, it is plausible that differential responses to different generations of ALK TKIs may be related to the underlying biology of the tumor.

The detected *KDR-Q472H* alteration is usually not considered as a targetable driver, its role in tumorigenesis is possibly through enhanced vascularization [21,22]. The tumor harbored a *TP53-R273H* mutation, which is a common pathogenic *TP53* variant [23,24,25,26]. Based on preclinical experiments, *TP53-R273H* can cause resistance to cisplatin and doxorubicin [14,15], which prompted the immediate switch to targeted TKI therapy from the started first-line cisplatin+gemcitabine/pemetrexed treatment. It has been well established that the presence of *TP53* mutations indicates higher risk in *ALK*-positive NSCLC [3,13,16]. Rapid progression on first-line crizotinib treatment is in line with published evidence demonstrating poor efficacy of this drug in *TP53*-mutant ALK-positive tumors [3,13,16], thus our results further confirm that *TP53* status should be taken into consideration when selecting ALK-inhibitor treatment for personalized therapies. Moreover, the second-generation ALK inhibitor, ceritinib, achieved tumor response for well over two years, then, following progression, the third-generation ALK inhibitor, lorlatinib also achieved disease control for the duration of the treatment. It has been proposed that future studies are required to determine whether *TP53* mutations present a similarly negative prognosis for next-generation ALK inhibitors as for crizotinib [16]. Our results thus also warrant future studies to analyze whether this observation can be conceptualized.

In conclusion, the presented case strongly underscores the importance of continuous personalized treatment decisions based on molecular diagnostics and monitoring results of *ALK*-positive NSCLC cases. In a *TP53*-mutant background, crizotinib has proved to be ineffective, whereas switching TKI generations may overcome treatment exhaustion even in the absence of evident ALK-dependent resistance mechanisms.
